# Kinetic and Thermodynamic Studies of Lysozyme Adsorption on Cibacron Blue F3GA Dye-Ligand Immobilized on Aminated Nanofiber Membrane

**DOI:** 10.3390/membranes11120963

**Published:** 2021-12-07

**Authors:** Ai Hsin, Su-Chun How, Steven S.-S. Wang, Chien Wei Ooi, Chen-Yaw Chiu, Yu-Kaung Chang

**Affiliations:** 1 Department of Chemical Engineering, National Taiwan University, Taipei 10617, Taiwan; luihsin2015@gmail.com; 2 Department of Chemical Engineering and Biotechnology, Tatung University, Taipei 104, Taiwan; hg43113@gmail.com; 3 Chemical Engineering Discipline, School of Engineering, Monash University Malaysia, Jalan Lagoon Selatan, Bandar Sunway 47500, Malaysia; ooi.chien.wei@monash.edu; 4 Department of Chemical Engineering, Graduate School of Biochemical Engineering, Ming Chi University of Technology, New Taipei City 243303, Taiwan; chenyaw@gmail.com

**Keywords:** dye affinity ligand, nanofibrous membrane, lysozyme, adsorption, kinetic, thermodynamic

## Abstract

The polyacrylonitrile (PAN) nanofiber membrane was prepared by the electrospinning technique. The nitrile group on the PAN nanofiber surface was oxidized to carboxyl group by alkaline hydrolysis. The carboxylic group on the membrane surface was then converted to dye affinity membrane through reaction with ethylenediamine (EDA) and Cibacron Blue F3GA, sequentially. The adsorption characteristics of lysozyme onto the dye ligand affinity nanofiber membrane (namely P-EDA-Dye) were investigated under various conditions (e.g., adsorption pH, EDA coupling concentration, lysozyme concentration, ionic strength, and temperature). Optimum experimental parameters were determined to be pH 7.5, a coupling concentration of EDA 40 μmol/mL, and an immobilization density of dye 267.19 mg/g membrane. To understand the mechanism of adsorption and possible rate controlling steps, a pseudo first-order, a pseudo second-order, and the Elovich models were first used to describe the experimental kinetic data. Equilibrium isotherms for the adsorption of lysozyme onto P-EDA-Dye nanofiber membrane were determined experimentally in this work. Our kinetic analysis on the adsorption of lysozyme onto P-EDA-Dye nanofiber membranes revealed that the pseudo second-order rate equation was favorable. The experimental data were satisfactorily fitted by the Langmuir isotherm model, and the thermodynamic parameters including the free energy change, enthalpy change, and entropy change of adsorption were also determined accordingly. Our results indicated that the free energy change had a negative value, suggesting that the adsorption process occurred spontaneously. Moreover, after five cycles of reuse, P-EDA-Dye nanofiber membranes still showed promising efficiency of lysozyme adsorption.

## 1. Introduction

Electrospun nanofiber membranes have been intensively researched in fields [1], such as tissue engineering [2], drug delivery [3], biosensing [4], antibacterial membranes [5], filtration and adsorptive membranes [6,7], and wastewater treatment [8].

Desirable features of nanofiber membranes intended for use in protein adsorption include low non-specific adsorption, high adsorption capacity, high adsorption rate, good biocompatibility, and high chemical/mechanical stability; these characteristics can be achieved by manipulation of chemical composition and synthesis operation that result in high porosity, large specific adsorption area, and good hydrophilicity of nanofiber membranes [6,9,10,11,12,13]. Polyacrylonitrile (PAN) membranes have superior mechanical strength and chemical stability [14,15], but their hydrophobic nature makes them less suitable for application in protein adsorption, as compared with the cellulose membranes that have good hydrophilicity [16,17]. As a result, introduction of the hydrophilic groups on the PAN membrane surface could combine the advantages offered by both hydrophilic and hydrophobic membranes.

Immobilization of dye ligand onto the PAN-based nanofiber membranes has been proven effective in selective adsorption of target proteins [11,12,13]. Among different types of dyes, Cibacron blue F3GA (abbreviated as CB, or known as reactive blue 2 dye) reactive dye is the one widely utilized in protein adsorption [15,18]. The advantages of using the biomimetic dyes as ligands for protein purification include cost-effectiveness, ease of immobilization, high binding capacity, and medium specificity [19]. In addition, the dye molecules can be conjugated via the hydroxyl and/or amino groups on the membrane surface.

Chicken egg white (CEW) is composed of a variety of proteins including ovalbumin (~54%), ovotransferrin (~12–13%), ovomucoid (~12–13%), and lysozyme (~3.5%). CEW is considered as the main source of lysozyme, which is a commercially valuable biocatalyst [20] with substantial potential in the food or pharmaceutical industries [21]. Lysozyme is commonly used as the target enzyme to purify from the complex CEW [11,12,13].

While the adsorption characteristics of lysozyme on the dyed nanofiber membranes have been investigated [11,12,13], the detailed mechanism of lysozyme adsorption on the dyed nanofiber membranes remains elusive. In this study, the behavior of lysozyme adsorption on the CB-dye ligand affinity PAN nanofiber membrane was analyzed from the thermodynamic and kinetic perspectives. In the first part, the electrospinning technique was used to fabricate the PAN nanofiber membranes. The nitrile groups on the PAN membrane surface were first converted into the carboxylic groups via the hydrolysis process. Next, ethylenediamine (EDA) molecules were covalently bonded to the membrane surface, followed by the immobilization of dye molecules onto the EDA-modified membranes (P-EDA) to produce the dye ligand affinity nanofiber membrane (P-EDA-Dye). In the second part, three different isotherms (i.e., Langmuir and Freundlich, and Temkin) models were used to evaluate the experimental data and obtain the thermodynamic parameters (changes in Gibbs free energy (Δ*G*°), enthalpy (Δ*H*°) and entropy (Δ*S*°)) [13]. In addition, the rate data of lysozyme adsorption onto the dye ligand affinity nanofiber membrane were acquired from the batch adsorption experiments conducted under different conditions and analyzed to examine the mechanism of adsorption process and identify the rate controlling step. Lastly, the repeated uses of dye-immobilized P-EDA nanofiber membranes were evaluated.

## 2. Materials and Methods

Polyacrylonitrile (PAN) yarn (*Mw* = 120,000 g/mol, containing 93% acrylonitrile and 7% vinylacetate) was obtained from Fortune Industries Inc. (Tao-Yuan, Taiwan). Polyethyleneterephthalate (PET) spunbond fabric (basis weight 15 g/m^2^, thickness 85 μm) was supplied from Freudenberg Far Eastern Spunweb Co. (Tao-Yuan, Taiwan). 1-(3-Dimethylaminopropyl)-3-ethylcarbodiimide hydrochloride (EDC), *N*-hydroxysuccinimide (NHS), 2-(N-Morpholino) ethane sulfonic acid hydrate (MES), *N*,*N*-dimethylacetamide (DMAc), and ethylenediamine dihydrochloride (EDA 2HCl) were obtained from Sigma-Aldrich (St. Louis, MO, USA). All other chemicals and solvents were used without further purification. Electrospinning device was purchased from Falco Tech Enterprise Co., Ltd. (New Taipei City, Taiwan). CB reactive dye (C_29_H_20_ClN_7_O_11_S_3_, *Mw* = 774.16 mol/g) was sourced from the First Chemical Manufacture Co., Ltd. (Taipei, Taiwan).

### 2.1. Preparation of PAN Nanofibrous Membrane

**Figure 1:**
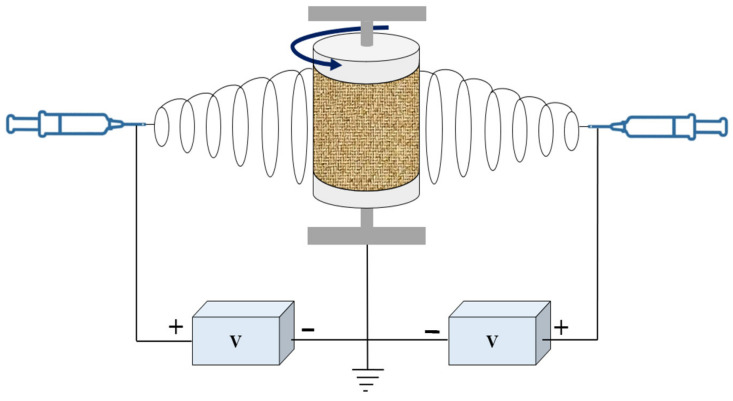
Schematic representation of the electrospinning device, including polymer solution stored in syringes, syringe pumps, DC high voltage power supplies, and cylindrical rotating collector.

The electrospinning process of PAN nanofiber membrane, as shown in Figure 1, was described previously described by Wang et al., 2018. PAN yarn (15 g) was dissolved in dimethylacetamide (DMAc) at 333 K under gentle stirring for 6 h to form a homogeneous solution. The PAN/DMAc solution was loaded into a springe and delivered to the tip of a 21-gauge stainless steel nozzle with a springe pump, which was connected to a power supply. The applied electric voltage was 26.5 kV; the distance between the needle tip and collector was 15.8 cm; the feed rate of the solution was 1 mL/h; the rotation rate of collector was 24 cm/s. The nozzle was moving along the y-axis (20 cm), and the frequency was 12 times/min. The PAN nanofibrous mat was deposited on PET spunbond nonwoven (basis weight 15 g/m^2^), which was pre-wrapped around the grounded steel drum. The PAN membrane was subjected to heat pressing process using a flat sandwich stainless steel plate at 373 K for 1 h. After the heat pressing process, the PAN-PET-PAN membrane became dense and entangled together.

### 2.2. Preparation of Weak Acidic Ion-Exchange Nanofiber Membrane

The PAN nanofiber membrane was first cut into 4.19 cm^2^ (i.d. 2.5 cm), before being placed in 3 M NaOH at 353 K for 17 min. After alkaline treatment, the resultant carboxylated nanofibrous membrane (P-COOH) was continuously washed with distilled water to remove the residue NaOH. The membrane was then treated with 0.1 M HCl, followed by drying in an oven at 333 K before use.

### 2.3. Preparation of Aminated PAN Nanofiber Membrane

A piece of P-COOH nanofiber membrane was submerged into 5 mL of 0.1 M MES solution containing 200 μmol EDC and EDA 2HCl (pH 6.0). The mixture was shaken at 100 rpm and 298 K for 3 h. Finally, the aminated nanofiber membrane (P-EDA) was taken out, washed with H_2_O to remove the free EDA, and then dried in an oven at 333 K. The amount of EDA on the membrane was determined by measuring the initial and final EDA concentrations in the liquid phase.

### 2.4. Immobilization of Dye onto Aminated Membrane

**Figure 2:**
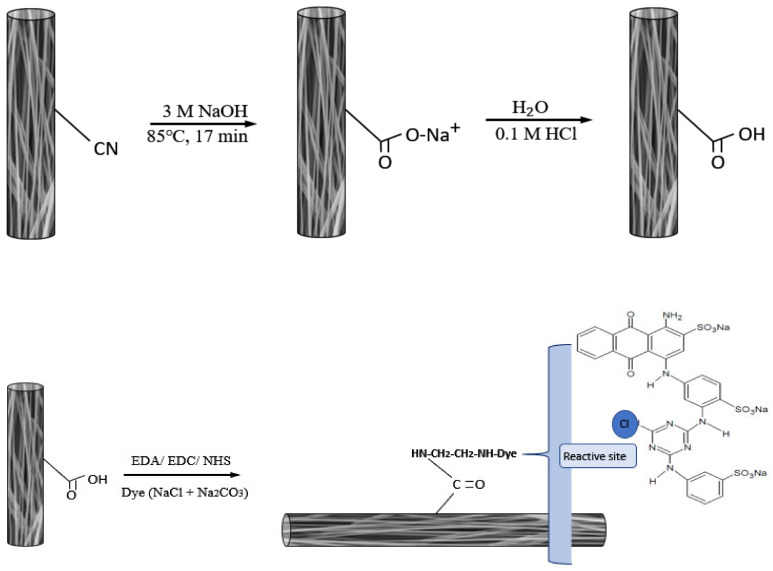
The chemical synthesis routes of P-COOH, P-EDA, and P-EDA-Dye nanofiber membranes.

A piece of P-EDA nanofiber membrane (approximately 30 mg, 4.19 cm^2^) was placed in a flask containing 0.5–4.0 CB dye mg/mL dye and 20% NaCl in 5 mL 0.1 M Na_2_CO_3_ at pH 11. The reaction mixture was then heated at 333 K for 6 h in a sealed flask. The dyed P-EDA nanofiber membrane (P-EDA-Dye) were thoroughly washed with a series of solutions (i.e., warm H_2_O, 10% ethanol, 1 M NaCl, and 6 M urea) until no dye molecule was detected in the washing solution. The dye concentration in solution was measured by spectrophotometer at 620 nm [11]. The immobilized dye on the P-EDA-Dye was determined by the difference of initial and final concentration of dye. Chemical synthesis route of P-COOH, P-EDA, and P-EDA-Dye nanofiber membrane are shown in Figure 2.

### 2.5. Effect of Operating Parameters on Lysozyme Adsorption

The adsorption of lysozyme from aqueous medium on the P-EDA-Dye nanofiber membrane was first studied using various buffer solutions, e.g., sodium acetate buffer (5 mL, 20 mM, pH 4–5), sodium phosphate buffer (5 mL, 20 mM, pH 6–8), and glycine NaOH buffer (5 mL, 20 mM, pH 9–10). The initial concentration of lysozyme in each buffer solution was 0.8 mg/mL. The adsorption experiments were performed at 298 K and 100 rpm for 3 h. After this period had elapsed, the amount of lysozyme adsorbed on the membrane (mg lysozyme/g membrane) was determined by measuring the initial and equilibrium concentrations of lysozyme in the adsorption buffer. Different sets of batch adsorption studies were performed to investigate the effects of EDA concentration (20–200 μmol/mL), dye (0.1–4.0 mg/mL), sodium chloride concentration (0.1–1.0 M) during the adsorption process at 298 K. The effect of temperature on lysozyme adsorption was studied in 20 mM sodium phosphate buffer (pH 7.5, 5 mL) with various lysozyme concentrations (0.1–1.0 mg/mL) at different temperatures (278–308 K). The lysozyme concentration in liquid phase was determined using the absorbance measured at 280 nm and the extinction coefficient of 2.65 (mL/mg·cm) [22]. All the experiments were performed in triplicate with standard deviation less than 5%. The data are presented as the mean values determined from the independent experiments.

### 2.6. Adsorption Studies

In the kinetic adsorption experiment, various parameters were investigated in batch mode, including adsorption pH (4–11), immobilized dye concentration (0.5–4.0 mg/mL), EDA concentration used in nanofiber membrane fabrication (100–1000 μmol/5 mL), and temperature of adsorption experiments (278–308 K). In brief, a piece of P-EDA-Dye nanofiber membrane (approximately 30 mg) was added to a flask containing 5 mL of lysozyme solution. The flasks were sealed and placed in a shaker at 100 rpm for 3 h. At the designated time *t*, the samples of supernatants were collected from the flask for the determination of lysozyme concentration. The amount of adsorbed lysozyme on the membrane phase (mg lysozyme/g membrane) at time *t* was calculated according to the following equation:

(1) $$q_{t}=\frac{v\left(C_{o}-C_{t}\right)}{w}$$

where *C_o_* and *C_t_* (mg lysozyme/mL solution) are the initial lysozyme concentration before adsorption process and the lysozyme concentration at time *t* of adsorption process, respectively. *q_t_* (mg lysozyme/g membrane) is the concentration of lysozyme in the membrane solid phase at time *t*. *v* is the volume of aqueous phase (mL) and *w* is the weight of membrane (g). For the equilibrium studies in batch mode, a range of concentration of lysozyme (0.1–1.0 mg/mL) was made up in 5 mL of 20 mM sodium phosphate buffer, pH 7.5 in separate flasks. The flasks were placed in a shaker at different temperatures (278–308 K) at 100 rpm for 3 h. The amount of adsorbed lysozyme on the membrane phase at equilibrium was calculated by Equation (1).

### 2.7. Adsorption Kinetic Models

#### 2.7.1. Pseudo First-Order Kinetic Model

A simple adsorption kinetic model, the pseudo first-order kinetic model, can be described by Equation (2) [23,24]:

(2) $$\frac{dq_{t}}{dt}=k_{1}\left(q_{1}-q_{t}\right)$$

Integration of Equation (2) results in Equation (3):

(3) $$ln\left(q_{1}-q_{t}\right)=lnq_{1}-k_{1}t$$

The linear plots of *ln(q_eq_−q_t_)* vs. *t* for different experimental conditions were used to determine the overall rate constant (*k*_1_). In this adsorption kinetic model, the transport of protein to the interface of membrane is assumed to be the rate-limiting step.

#### 2.7.2. Pseudo Second-Order Kinetic Model

The pseudo second-order kinetic model can be described by Equation (3) [23,24]:

(4) $$\frac{dq_{t}}{dt}=k_{2}{\left(q_{2}-q_{t}\right)}^{2}$$

Integration of Equation (4) yields Equation (5):

(5) $$\frac{t}{q_{t}}=\frac{1}{k_{2}\times q_{2}^{2}}-\frac{t}{q_{2}}$$

The overall rate constant (*k*_2_) and equilibrium binding capacity (*q_eq_*) can be obtained from the intercept and slope of the plot of (*t/q_t_*) vs. *t*, respectively. When using this adsorption kinetic model, the surface reaction is assumed to be the rate-limiting step.

#### 2.7.3. Elovich Kinetic Model

The Elovich adsorption kinetic model can be described by the Elovich equation, which is shown below Equation (6) [25]:

(6) $$\frac{dq_{t}}{dt}=\alpha e^{-\beta q_{t}}$$

Elovich equation was derived via an integration of the rate equation using the same boundary conditions as the pseudo first- and second-order equation.

(7) $$q_{t}=\frac{1}{\beta }ln\left(\alpha \beta \right)+\frac{1}{\beta }ln\left(t\right)$$

where the parameter *α* is the initial sorption rate (mg/g·min) and the parameter *β* is related to the extent of surface coverage and activation energy for chemisorption (g/mg).

#### 2.7.4. Intra-Particle Diffusion Model

An intra-particle diffusion-based model, which can be described by Equation (8), was used to investigate the mechanism of the adsorption of lysozyme on the P-EDA-Dye nanofiber membrane [22].

(8) $$q_{t}=k_{i}\times t^{0.5}+I$$

where *k_i_* is the intra-particle diffusion rate constant, mg/g·min^0.5^. *k_i_* values under different conditions were determined from the slopes of straight-line portions of the respective plots.

In general, the adsorption mechanism could involve several steps in which one or any of their combination would control the rate of adsorption. There are three steps involved: (1) the mass transfer across the external boundary layer film of liquid surrounding the outside of the particle, (2) the adsorption at a site on the internal/external surface, which is often assumed to be very rapid, (3) the diffusion of target molecules to an adsorption site by diffusion either through the liquid-filled pores or the solid surface.

### 2.8. Adsorption Equilibrium Studies

#### 2.8.1. Langmuir Isotherm Model

Adsorption isotherms studies have been conducted to characterize the adsorption of lysozyme by the membrane [13]. The adsorption isotherm curves reflect the binding capacity and binding strength of the adsorbent. Therefore, it is important to establish the appropriate correlations for the equilibrium isotherm studies. To describe the adsorption of lysozyme, a typical Langmuir isotherm curve was generated using the Langmuir equation shown in Equation (9) [22,23].

(9) $$q=\frac{q_{max}\times C}{K_{d}+C}$$

where *C* (mg lysozyme/mL solution) is the equilibrium concentration of lysozyme in the aqueous phase. *q* (mg lysozyme/g-membrane) is the equilibrium lysozyme concentration in the membrane solid phase. *q_max_* is the maximum binding capacity of membrane for lysozyme (mg lysozyme/g) and *K_d_* is the dissociation constant of lysozyme-membrane complex (mg lysozyme/mL solution). The Langmuir constants, *q_max_* and *K_d_*, were evaluated from the corresponding semi-reciprocal plot (*C/q* vs. *C*) of the linear Equation (10).

(10) $$\left(\frac{C}{q}\right)=\left(\frac{C}{q_{max}}\right)+\left(\frac{K_{d}}{q_{max}}\right)=\left(\frac{C}{q_{max}}\right)+\left(\frac{1}{q_{max}\cdot K_{L}}\right)$$

The value of *K_d_* (mg/mL) denotes the binding strength between lysozyme and membrane matrix and is considered an inverse measure of the equilibrium association constant (*K_L_*, mL/mg) for the binding between the membrane and lysozyme.

#### 2.8.2. Freundlich Isotherm Model

The Freundlich model assumes that the biomolecule adsorption takes place on a heterogeneous surface through a monolayer adsorption [22,23]. This model can be described by the following equation:

(11) $$q=K_{F}C^{\frac{1}{n}}$$

where *K_F_* and *n* can be related to binding capacity and adsorption intensity, respectively, and are determined from the straight-line plots of *ln(q)* vs. *ln(C)* for the adsorption of lysozyme as described in Equation (12).

(12) $$lnq=lnK_{F}+\frac{1}{n}lnC$$

#### 2.8.3. Temkin Isotherm Model

The Temkin isotherm assumes that the heat of adsorption decreases linearly rather than logarithmically due to the adsorbate/adsorbent interaction, as implied in the Freundlich equation. The linear form of Temkin isotherm is given in the following equation [22,23]:

(13) $$q=\frac{RT}{b}ln\left(K_{T}\right)+\frac{RT}{b}ln\left(C\right)$$

where *b* is related to the heat of adsorption (J/mol), *R* is gas constant (8.314 J/mol/K), *T* is the temperature (K), and *K_T_* is equilibrium binding constant (mL/g). These constants were determined from, a plot of *q* versus *ln(C)*.

## 3. Results and Discussion

### 3.1. Physical Properties of Nanofiber Membranes

**Figure 3:**
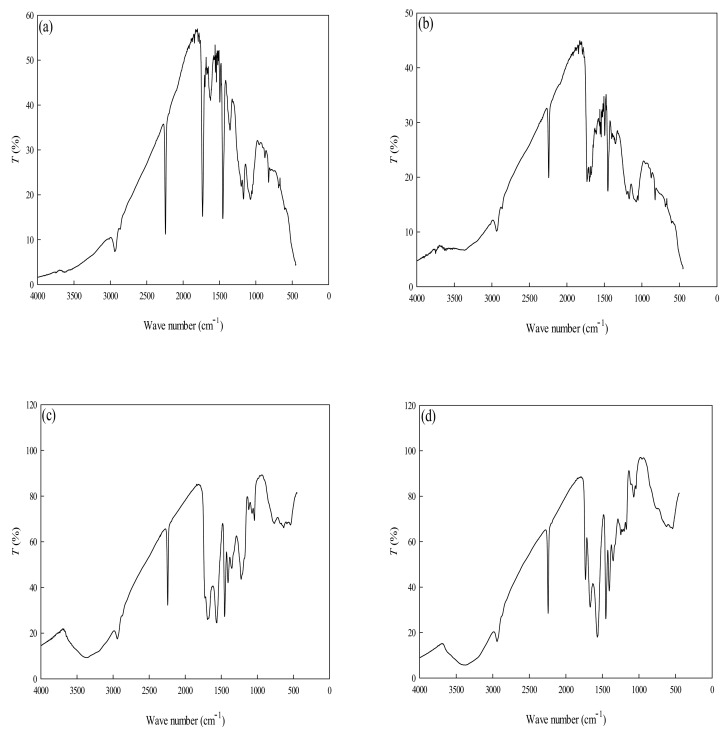
FTIR spectra of (**a**) PAN, (**b**) P-COOH, (**c**) P-EDA, and (**d**) P-EDA-Dye nanofiber membranes.

FTIR spectra of PAN, P-COOH, P-EDA, and P-EDA-Dye nanofiber membranes show numerous peaks which indicate their chemical structures (Figure 3). The broad peak appeared between 3650–3200 cm^−1^ in P-COOH, P-EDA, P-EDA-Dye nanofiber membranes are attributed to the -OH stretch from the alcohols and phenols groups [12,13]. The peak detected between 2260–2240 cm^−1^ (PAN, P-COOH, P-EDA, and P-EDA-Dye nanofiber membranes) are vibrational signal generated from cyanate group (-CN) [26]. Furthermore, three absorption peaks at 3550–3300 cm^−1^ (-OH stretch), 1640–1560 cm^−1^ (-N=N-), and 820 cm^−1^ (-COO) are the manifestations from the absorption of -NH_2_ groups available on the material [13]. FTIR analysis only provides the qualitative insights on these materials. Hence, to quantify the important chemical contents such as amine and carboxyl contents in the nanofiber membranes, the measurement of functional groups concentration was performed based on the adsorption of the respective dyes (e.g., AO7 and TBO) [27]. The amine (-NH_2_) groups on the P-EDA nanofiber membrane were ~392 μmol/g, based on the reaction of the negatively charged AO7 dye with the positive surface functional group (-NH_3_^+^). Similarly, the positively charged TBO dye reacted with the negative surface ions (-COO^−^) whereby the number of carboxyl groups on the P-COOH nanofiber membrane was ~423 μmol/g.

**Figure 4:**
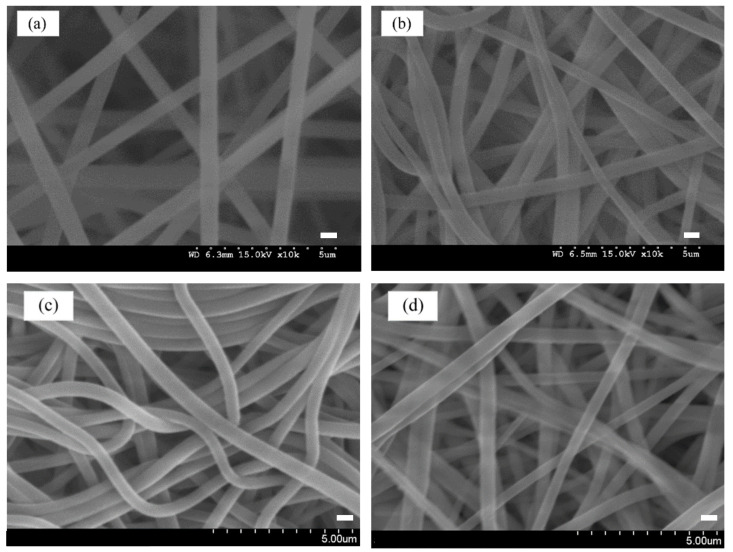
SEM images of (**a**) PAN, (**b**) P-COOH, (**c**) P-EDA, and (**d**) P-EDA-Dye nanofiber membranes. Bar scale: 500 nm.

The surface morphological attributes of PAN, P-COOH, P-EDA, and P-EDA-Dye nanofiber membranes are shown in Figure 4a,d, respectively. The range of fiber diameters is 300–600 nm. In the pristine form (PAN), the nanofiber membrane showed neat and tubular structures, whereby its morphological structure was similar to some reported in the literature [12,13]. After going through alkaline (NaOH) and acid (HCl) treatments, the structure of the resultant P-COOH nanofiber membrane showed randomly overlapped of fibers (Figure 4b). Compared with PAN nanofiber membrane, P-COOH nanofiber membrane possessed more arbitrary orientation of fibers. P-EDA nanofiber membrane also shared the similar structures as P-COOH nanofiber membrane. After the reaction with CB dye, the P-EDA-Dye nanofiber membrane did not experience drastic structural change. However, as shown in Figure 4d, bright spots were detected from the SEM imaging. After the modification process with dye, the surface characteristics of P-EDA-Dye nanofiber membrane exhibited electrostatic properties from the reaction.

### 3.2. Effect of Operating Parameters on the Lysozyme Adsorption

**Figure 5:**
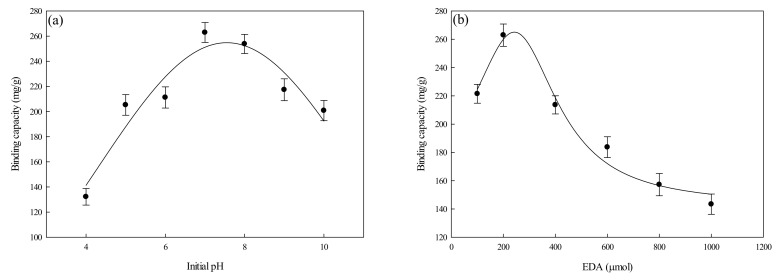

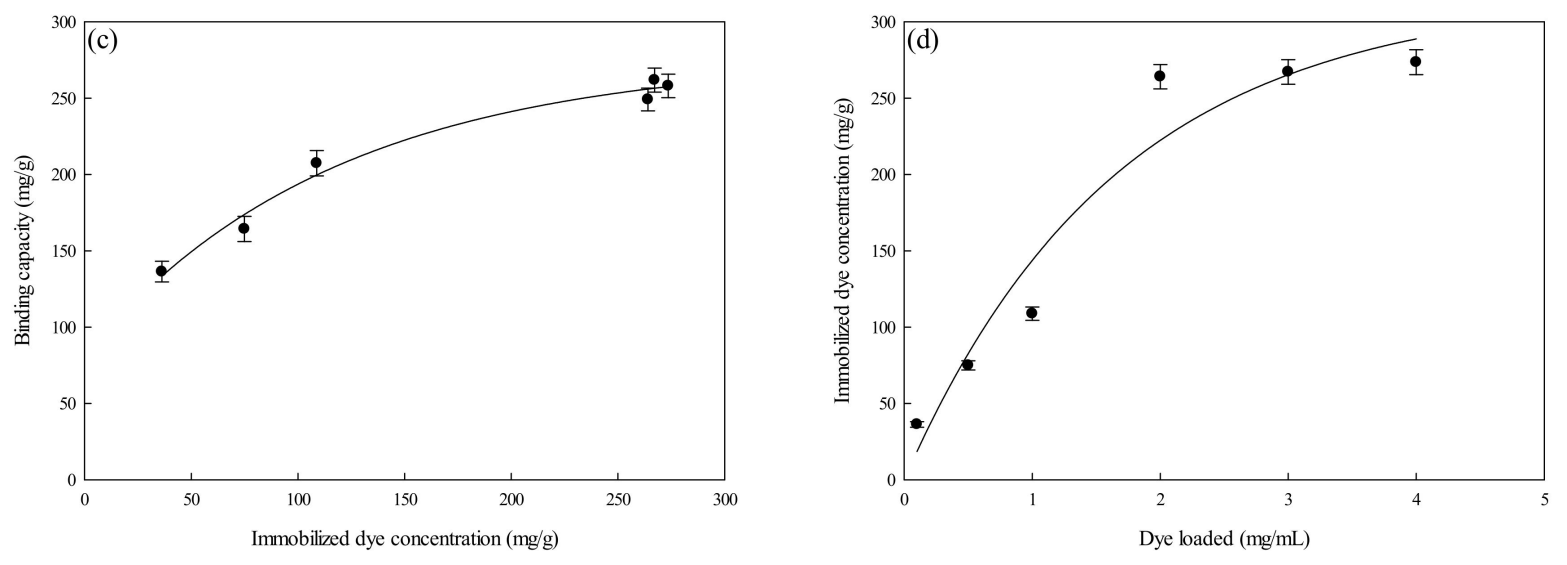
Effects of operating parameters on the binding capacity of P-EDA-Dye nanofiber membranes for lysozyme: (**a**) adsorption pH, (**b**) concentration of EDA coupled on P-COOH nanofiber membrane, (**c**) dye concentration immobilized on P-EDA-Dye nanofiber membrane, and (**d**) dye immobilization concentration vs. dye loaded.

The binding capacity of the P-EDA-Dye nanofiber membrane for lysozyme under various operating parameters (e.g., adsorption pH, concentration of coupled EDA, and immobilized dye concentration) was investigated. The results are shown in Figure 5a,d.

#### 3.2.1. Effect of Initial Adsorption pH

Studies of the adsorption of lysozyme by P-EDA-Dye nanofiber membrane were carried out in the adsorption pH ranging from 4 to 10. As observed from Figure 5a, the varying adsorption pH values from 4 to 10 have significant impact on the binding capacity of P-EDA-Dye nanofiber membrane. The maximum binding capacity of P-EDA-Dye nanofiber membrane for lysozyme was approximately at pH 7.5 (~262.93 mg/g). The P-EDA-Dye nanofiber membrane showed lower binding capacities below pH 7 and above pH 8. As shown in Figure 5a, this trend of the binding capacity may be attributed to the asymmetric distribution of surface charge on the lysozyme molecule (p*I* = 11). Here, the positively charged groups may be present in specific region of the lysozyme molecule, although there exists more positively charged amino groups under a lower pH condition [28]. Similar results were reported previously [12,13]. Although the adsorption process is mainly governed by the charge distribution on lysozyme, it is worth noting that the binding capacity of P-EDA-Dye nanofiber membrane could not be solely explained by the net charge concept.

As the adsorption pH was increased to 10 (close to the p*I* value of lysozyme), the binding capacity of P-EDA-Dye nanofiber membrane decreased to ~200.81 mg/g, which may be due to less positively charged groups on the lysozyme. This contributes to a less pronounced electrostatic effect on the positively charged lysozyme and the negatively charged P-EDA-Dye nanofiber membrane (3 units of SO_3_Na). Our results suggested that the optimal pH for lysozyme adsorption on P-EDA-Dye nanofiber membrane is pH 7.5.

#### 3.2.2. Effect of Concentration of Coupled EDA

The EDA-modified nanofiber membrane contains a high degree of amine groups serving as a weak cation (-NH_2_) and/or weak anion (-NH_3_^+^) exchanger. The P-EDA nanofiber membrane may either repel or attract protein molecules depending on their p*I* values and the degree of positive charge on their surface. In addition, the adsorption process of lysozyme with polyelectrolytic characteristics may be controlled by the molecular size and flexibility of lysozyme, diffusion behavior of lysozyme in both bulk solution and membrane pores, and strength of hydrophilic and/or hydrophobic forces existing between the lysozyme and the membrane [13]. Hence, the effect of the amount of EDA (100–1000 μmol, 5 mL) used to couple with P-COOH nanofiber membrane was investigated. As described previously [13], the coupling pH of the selected EDA was 6 by EDC-mediated immobilized method. After the coupling reaction, the residual amine groups of an activated P-EDA nanofiber membrane can be easily modified with reactive dye molecule. Figure 5b shows the effects of concentration of EDA coupled onto the P-COOH nanofiber membrane used for the adsorption of lysozyme. When the EDA coupling amount were raised from 100 to 1000 µmol, the binding capacity of P-EDA-Dye for lysozyme reached a maximum value (262.93 mg/g) when the concentration of coupled EDA was 200 μmol. Therefore, the optimal mole number of EDA used in the preparation of P-EDA-Dye nanofiber membrane was 200 μmol.

#### 3.2.3. Effect of Dye Concentration on P-EDA-Dye Nanofiber Membrane

The density/concentration of dye immobilized onto the P-EDA-Dye nanofiber membrane is considered as an important factor affecting the adsorption efficiency of proteins [12,13]. Figure 5c shows the relationship between lysozyme binding capacity of P-EDA-Dye nanofiber membrane and dye concentration immobilized on P-EDA-Dye nanofiber membrane (0.1–2.0 mg/mL). The difference in lysozyme binding capacity of P-EDA-Dye nanofiber membranes immobilized with different concentrations of dye may be due to the changes in the (i) mass of the immobilized dye molecule; (ii) charge distribution and arrangement of the dye molecule; or (iii) the protein–dye interactions via ionic, hydrophobic, or specific interaction) [13]. As shown in Figure 3c, the maximum binding capacity of P-EDA-Dye nanofiber membrane for lysozyme was 261.83 mg/g when 3.0 mg/mL of dye was loaded, which corresponded to the mass of the immobilized dye of 267.19 mg/g (345.14 μmol/g). Hence, it was suggested that the optimal mass of immobilized dye on the P-EDA-Dye nanofiber membrane was 22.7 mg/g.

By varying the immobilized dye density (i.e., 36.26–273.58 mg dye/g membrane), the binding capacity of P-EDA-Dye nanofiber membrane for lysozyme ranged in between 136.37 mg/g and 258.05 mg/g, as shown in Figure 5d. It was found that the binding capacity of P-EDA-Dye nanofiber membrane for lysozyme was significantly affected by the immobilized dye concentration. An increase in the dye loading (>0.5 mg/mL) gave rise to a higher binding capacity of P-EDA-Dye nanofiber membrane for lysozyme. As a result, the degree of steric hindrances between dye and lysozyme molecules on the P-EDA-Dye nanofiber membrane was not viewed as an important parameter for the binding of lysozyme.

### 3.3. Kinetic Studies of Lysozyme Adsorption

#### 3.3.1. Effect of Lysozyme Concentration

**Figure 6:**
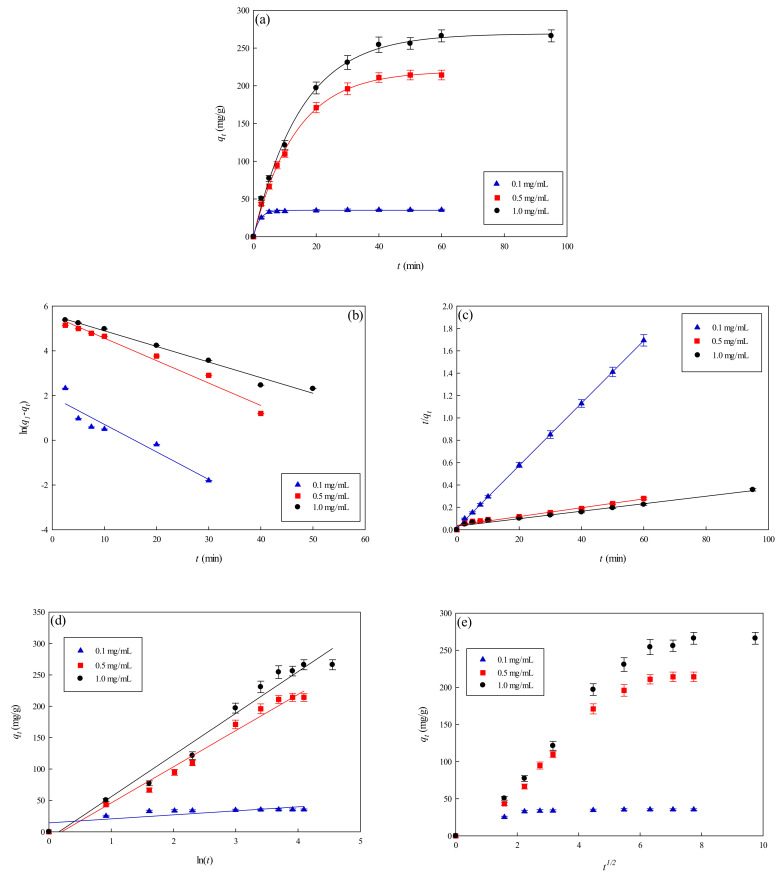
Adsorption rate at varying concentrations of lysozyme by P-EDA-Dye nanofiber membrane. (**a**) Kinetic curves at varying concentrations of lysozyme, (**b**) pseudo first-order plot of ln(*q*_1_*−q_t_)* against *t*, (**c**) pseudo second-order plot of *t/q_t_* against *t*, (**d**) Elovich model plot of *q_t_* against ln *t*, and (**e**) intra-particle diffusion model plot of *q_t_* against *t*^0.5^.

Figure 6a depicts the rate of adsorption of lysozyme onto the P-EDA-Dye nanofiber membrane at varying lysozyme concentrations (e.g., 0.1, 0.5, and 1.0 mg/mL) as a function of time. A relatively higher adsorption rate was observed when a higher initial lysozyme concentration was used in the beginning of the adsorption process. The initial rate was observed to increase from 10.07 to 20.23 mg lysozyme/min·g as the initial lysozyme concentration increased from 0.1 to 1.0 mg lysozyme/mL solution. Given that the adsorption equilibrium was achieved within 40 min, lysozyme molecules have a rather high affinity toward the active sites of P-EDA-Dye nanofiber membrane. The binding capacity at the equilibrium stage increased from 35.26 to 254.45 mg lysozyme/g with an increase in the initial lysozyme concentrations used. There is a positive correlation between the lysozyme concentration in adsorption medium and the adsorption rate. This trend could be explained using the concept of concentration difference between the liquid and solid phases. A greater concentration difference across the liquid and solid phases gave rise to a larger driving force, which can overcome all mass transfer resistances, resulting in the higher probabilities of collision between the membrane adsorption sites and lysozyme molecules; thus, it leads to a higher binding capacity at a higher initial lysozyme concentration used. Lysozyme is a nearly spherical protein with size 30Å × 30Å × 45Å, as reported by Whitley et al. (1989) [29]. As the dye-immobilized P-EDA-Dye nanofiber membrane contains pores with an average size of ~0.45 µm, lysozyme molecule diffusion through the channels and/or internal pores of the membrane can easily penetrate to the binding sites below the surface of P-EDA-Dye nanofiber membrane.

#### 3.3.2. Effect of NaCl Concentration

**Figure 7:**
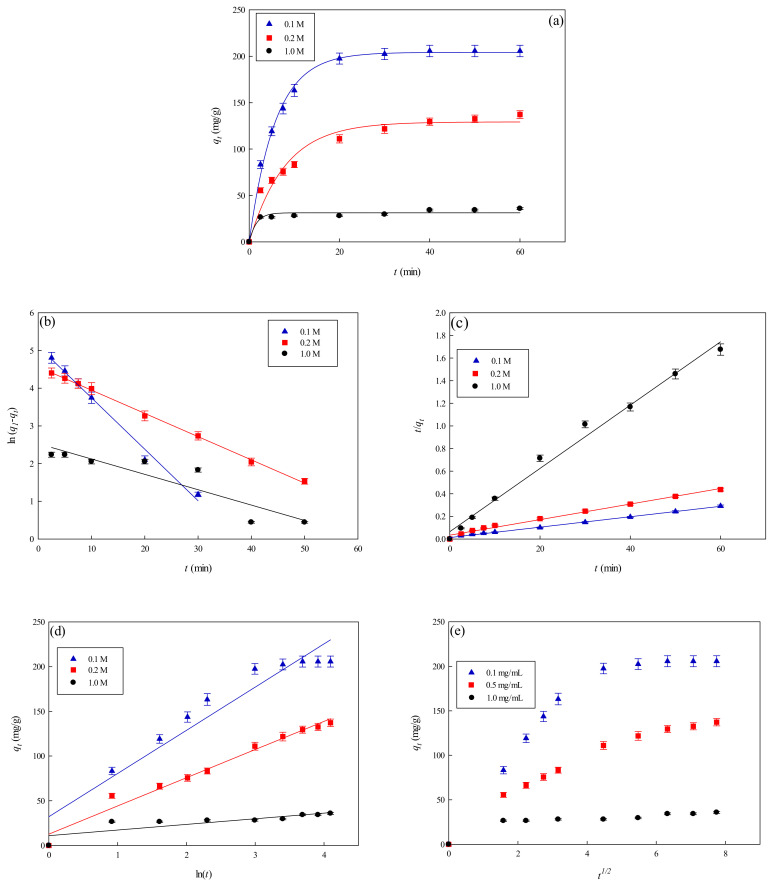
Adsorption rate at varying concentrations of NaCl by P-EDA-Dye nanofiber membrane. (**a**) Kinetic curves at varying concentrations of NaCl, (**b**) pseudo first-order plot of ln(*q*_1_−*q_t_*) against *t*, (**c**) pseudo second-order plot of *t/q_t_* against *t*, (**d**) Elovich model plot of *q_t_* against ln *t*, and (**e**) intra-particle diffusion model plot of *q_t_* against *t*^0.5^.

Since large amounts of salts are generally utilized in the elution process of the adsorbed lysozyme from the membrane [12,13], the effects of ionic strength on adsorption must be evaluated. Figure 7a shows the effects of ionic strength on the lysozyme adsorption by the P-EDA-Dye nanofiber membrane. The amount of eluted lysozyme was close to the maximum value at approximately 30 min. The binding capacity of P-EDA-Dye nanofiber membrane at the equilibrium stage decreased from 205.72 to 35.82 mg lysozyme/g with an increase in the NaCl concentrations from 0.1 to 1.0 M. An increase in NaCl concentration in the adsorption medium leads to a decrease in the binding capacity of P-EDA-Dye nanofiber membrane. Experimental results indicated that the increasing solution ionic strength decreased the adsorption of lysozyme by the P-EDA-Dye nanofiber membrane. The results indicated that the binding capacity decreased significantly upon addition of NaCl, implying that lysozyme attachment onto the surface of P-EDA-Dye nanofiber membrane may be mainly controlled by electrostatic interactions. However, other binding interactions (e.g., hydrophobic, hydrogen-bonding, and specific binding) may be present between lysozyme and P-EDA-Dye nanofiber membrane.

#### 3.3.3. Effect of Operating Temperature

**Figure 8:**
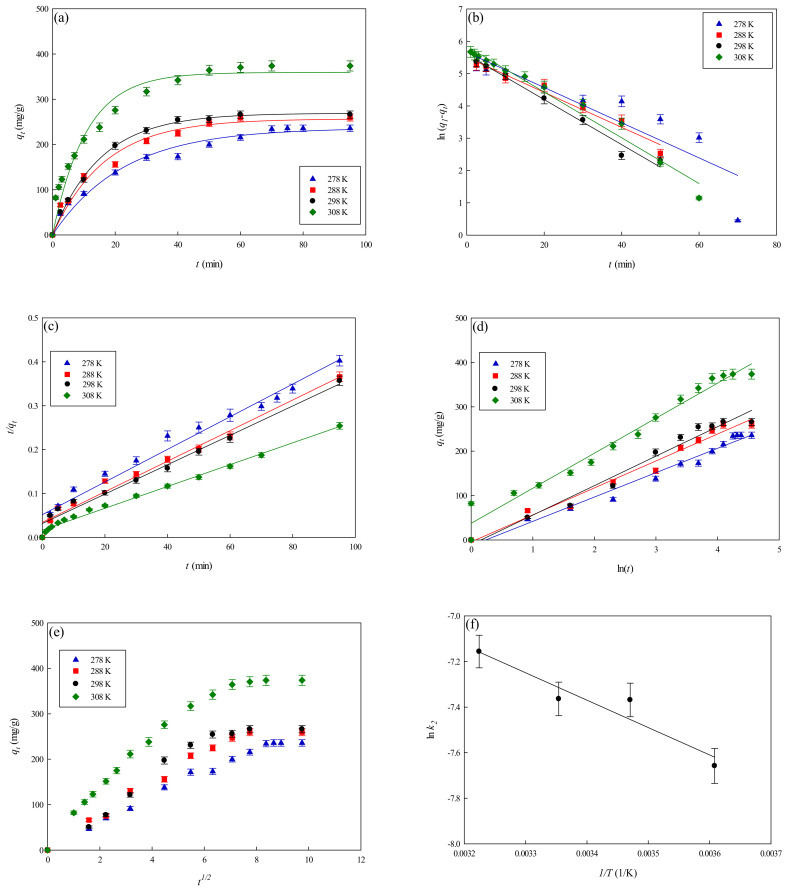
Adsorption rate of lysozyme at varying temperatures by P-EDA-Dye nanofiber membrane. (**a**) Kinetic curves at varying temperatures (278 K–308 K), (**b**) pseudo first-order plot of ln(*q*_1_−*q_t_*) against *t*, (**c**) pseudo second-order plot of *t*/*q_t_* against *t*, (**d**) Elovich model plot of q_t_ against ln(*t*), (**e**) intra-particle diffusion model plot of *q_t_* against *t*^0.5^, and (**f**) Arrhenius plot of ln *k*_2_ against 1*/T* for the adsorption of lysozyme on the P-EDA-Dye nanofiber membrane.

The rate of adsorption of lysozyme onto the P-EDA-Dye nanofiber membrane was investigated at varying temperatures from 278 K to 308 K, and the results are shown in Figure 8a. It was found that the initial adsorption rate increased with an increase in the temperature. The values of *q_eq,exp_* increased from 235.96 to 373.73 mg lysozyme/g when the temperature raised from 278 K to 308 K. Since the adsorption rate of hydrophobic or specific affinity interaction would increase as the temperature increases [11], the mechanism of lysozyme adsorption in our case may involve multi-binding interactions between lysozyme and P-EDA-Dye nanofiber membrane. In addition, an increase in temperature caused the increase of binding force between lysozyme and membrane. Thus, the binding capacity at a higher temperature was greater than that at a lower temperature. Similar results were also observed in the adsorption of lysozyme on different membrane systems [13].

#### 3.3.4. Kinetic Model Analysis

To investigate the governing mechanism of the adsorption process, pseudo first-order and pseudo second-order kinetic models were used to analyze the dynamic experimental data and to determine the adsorption rate constants. Due to the cationic characteristics of P-EDA-Dye nanofiber membrane (with -SO_3_^−^ groups), the mechanism of adsorption of lysozyme may be an ion exchange process. The dyed nanofiber membrane, *Na^+^(P-EDA-Dye)^−^*, has the single *Na^+^* ion balancing the charge of one dye immobilized composite membrane in the membrane framework, lysozyme molecule (namely *P-NH_3_^+^*) is the positively charged group at pH adsorption pH 7.5, and *(P-EDA-Dye)^−^-(NH_3_^+^-P)* represents charge–charge interactions between the immobilized lysozyme and dyed membrane. Comparison of the experimental adsorption capacities of the P-EDA-Dye nanofiber membrane for lysozyme and their theoretical estimates are presented in Table 1 and Figure 6, Figure 7 and Figure 8. Apparently, the pseudo first-order kinetic model (Figure 6b, Figure 7b and Figure 8b) gave lower correlation coefficients, indicating that this model is less suitable for delineating the adsorption of lysozyme onto the membrane. On the contrary, the higher correlation coefficients (R^2^ > 0.98) were obtained when using the pseudo second-order kinetic model (Figure 6c, Figure 7c and Figure 8c), suggesting that the pseudo second-order kinetic model agreed well with the experimental data. Our results showed that the pseudo second-order kinetic model, which assumed that that the rate-limiting step was the surface reaction, could better describe the mechanism of lysozyme adsorption [13]. The calculated *q_eq,cal_* values (e.g., 266.18 mg/g at 298 K) were found to be close to the experimental *q_eq,exp_* values (e.g., 268.49 mg/g, 298 K).

The temperature dependence of overall rate constant (*k*_2_) was reported to follow the Arrhenius equation (Equation (14)) [30].

(14) $$k_{2}=A_{a}e^{\frac{-E_{a}}{RT}}$$

The results were illustrated as an Arrhenius plot in Figure 8d. From the experiment data, the overall activation energy (*E_a_*) for lysozyme adsorption onto the P-EDA-Dye nanofiber membrane was ~2389.37 cal/mol. The overall rate constant, *k*_2_, was found to increase from 2.67 × 10^−4^ to 3.71 × 10^−4^ mg lysozyme/min g when the temperature increases from 278 K to 308 K in the adsorption of lysozyme.

Table 1 lists the kinetic constants obtained from the Elovich equation. The coefficients of determination (*R*^2^) are relatively high (approximately 0.97), and the results are shown in Figure 6d, Figure 7d and Figure 8d. It was found that the Elovich model was suitable for describing the adsorption kinetic of lysozyme on the P-EDA-Dye nanofiber membrane. Reports indicated that the Elovich model better describes the adsorption process on the highly heterogeneous surfaces and surface reaction, or chemisorption serves as the dominant phenomenon [24]. While the Elovich model is useful in describing the adsorption on the highly heterogeneous membrane adsorbers, it does not provide any mechanistic insight. Our fitting results showed that the adsorption behavior could be properly described by the pseudo second-order kinetic model, implying that the chemisorption mechanism may play an important role in the adsorption of lysozyme onto the nanofiber membrane. The adsorption of lysozymes onto the P-EDA-Dye nanofiber membrane took place most likely via the surface exchange reactions until the surface functional sites were fully occupied, thereafter lysozyme molecules diffused into the fiber pores for further reactions.

As shown in Figure 6e, Figure 7e and Figure 8e for the fitting with intra-particle diffusion model, the plot of *q_t_* versus *t*^0.5^ at different temperatures presented multi-linearity relation, indicating more than one process affecting the lysozyme adsorption. The *k_i_* values under different operating conditions were calculated from the slopes of the straight-line portions of the respective plots. The plot of *q_t_* versus *t*^0.5^ presents the multi-linearity, indicating that two or more pore diffusion steps occur in the adsorption process [22,23]. Moreover, the lines did not pass through the origin, indicating that some degrees of external diffusion or boundary layer diffusion may control the adsorption process; in other words, the pore diffusion or intra-particle diffusion may not be the only rate-controlling step, but there exist other processes controlling the rate of adsorption.

As can be seen from Figure 6e, Figure 7e and Figure 8e, there are three different linear regions. The values of *k_i_* and *I* (intercept) obtained from each stage linear regression analysis are listed in Table 1. The first linear region indicates the external diffusion step. The second linear region is related to the micro-pore diffusion step. The third linear region is associated with the equilibrium adsorption stage. Overall, a multi-stage kinetic characteristics was exhibited in the adsorption of lysozyme by the P-EDA-Dye nanofiber membrane. It was observed that the order of adsorption rates was the first linear portion (*k_i,_*_1_) > the second linear portion (*k_i,_*_2_). The changes in *k_i_* values could be attributed to the adsorption stages of the external diffusion and internal channel or micro-pore diffusion, respectively. From the pore size distribution, P-EDA-Dye nanofiber membrane has an average pore size of 0.45 µm and the pores are randomly distributed over the entire membrane surface. Hence, the multistage adsorption processes would be observed in these cases. The second portion of the plot seemed to refer to the channel diffusion into micro-pores as the rate-limiting step in the adsorption process on the membrane.

### 3.4. Equilibrium Isotherm Study (Results and Discussion)

#### 3.4.1. Thermodynamic Model Analysis

**Figure 9:**
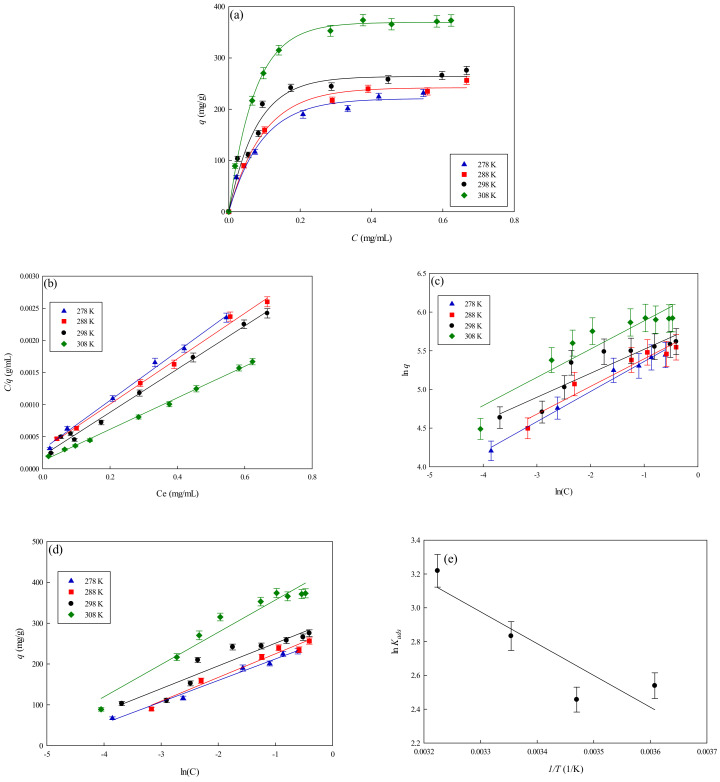
(**a**) Effect of temperature on the equilibrium isotherm curves for the adsorption of lysozyme on P-EDA-Dye nanofiber membrane, (**b**) Langmuir model plot of *C/q* against *C*, (**c**) Freundlich model plot of *ln*(*q*) against *ln*(*C*), (**d**) Temkin model plot of *q* against *ln*(*C*), and (**e**) Van ’t Hoff plot for the adsorption of lysozyme on P-EDA-Dye nanofiber membrane.

To explore the equilibrium behavior of lysozyme adsorption onto the nanofiber membranes, three isotherm models including Langmuir, Freundlich, and Temkin isotherms were used to fit against the equilibrium adsorption experiment data [22,23]. Figure 9a–e show the fitting of different adsorption isotherms with the experiment data of lysozyme adsorption on P-EDA-Dye nanofiber membranes at different temperatures (278–308 K). As shown in Table 2, the best correlation coefficient (*R*^2^ > 0.99) was obtained using the Langmuir isotherm, suggesting that the Langmuir isotherm was more adequate in fitting the experiment equilibrium data, as compared to the Freundlich model (*R*^2^ = 0.8541–0.9836) and Temkin model (*R*^2^ = 0.8918–0.9878). As shown in Table 3, for Langmuir isotherm model, the dissociation constant (*K_d_*) of P-EDA-Dye nanofiber membrane decreased from 0.0506 mg lysozyme/mL solution to 0.0845 mg lysozyme/mL solution. As the temperature rose from 278 to 308 K, the maximum binding capacity (*q_max,cal_*) of P-EDA-Dye nanofiber membrane increased from 262.19 to 407.66 mg/g. The observed elevation in binding capacity may be attributed to the chemical interaction between lysozyme and membrane, or the production of some new binding sites at higher temperatures. In comparison to P-CS-Dye membranes (i.e., dye immobilized on chitosan) [11], the P-EDA-Dye affinity nanofiber membrane has a higher capacity for lysozyme adsorption (275.50 mg/g), where *q_max,exp_* of P-CS-Dye nanofiber membrane was 245.58 mg lysozyme/g at 298 K. The *K_d_* value decreased with an increase in temperature, indicating that the adsorption of lysozyme onto nanofiber membranes was favorable at a higher temperature.

#### 3.4.2. Calculation of Thermodynamic Parameters

To calculate the thermodynamic parameters (Δ*G*°, Δ*H*°, and Δ*S*°), lysozyme was adsorbed onto the P-EDA-Dye nanofiber membrane at different temperatures ranging from 278 K to 308 K. The magnitudes of thermodynamic parameters (Δ*G*°, Δ*H*°, and Δ*S*°) were determined using the equations below [22,31]:

(15) $$\Delta G^{{}^{\circ}}=-RTlnK_{ads}$$

(16) $$ln(K_{ads})=ln(K_{o})-\left(\frac{\Delta H^{{}^{\circ}}}{R}\right)\times \frac{1}{T}$$

(17) $$\Delta S^{{}^{\circ}}=\frac{\Delta H^{{}^{\circ}}-\Delta G^{{}^{\circ}}}{T}$$

The dependence of the equilibrium association constant *K_ads_* on 1*/T* for the adsorption of lysozyme onto P-EDA-Dye nanofiber membrane, along with the corresponding Van ’t Hoff plot, are shown in Figure 9e. The slope of the plot of *ln K_ads_* vs. 1/*T* multiplied by *−R* (gas constant, 1.987 cal/mol·K) equals to $\Delta H^{{}^{\circ}}$ (corresponding to 3739.93 cal/mol). At a higher temperature, van der Waals interactions may dominate during the adsorption of lysozyme onto the membrane [13]. The estimates of the thermodynamic parameters of adsorption process at different temperatures are given in Table 3. The negative values for $\Delta G^{{}^{\circ}}$ suggested that the adsorption of lysozyme on the membrane was a spontaneous process. It was reported that $\Delta H^{{}^{\circ}}$ > 0 indicated that the adsorption process was endothermic, whereas $\Delta S^{{}^{\circ}}$ > 0 represented an increase in the total disorder of the system during the adsorption process. Here, the negative change in the apparent free energy indicated that the positive change in the apparent entropy contribution mainly dominated the adsorption process.

### 3.5. Repeated Use and Regeneration Capacity of P-EDA-Dye Nanofiber Membrane

**Figure 10:**
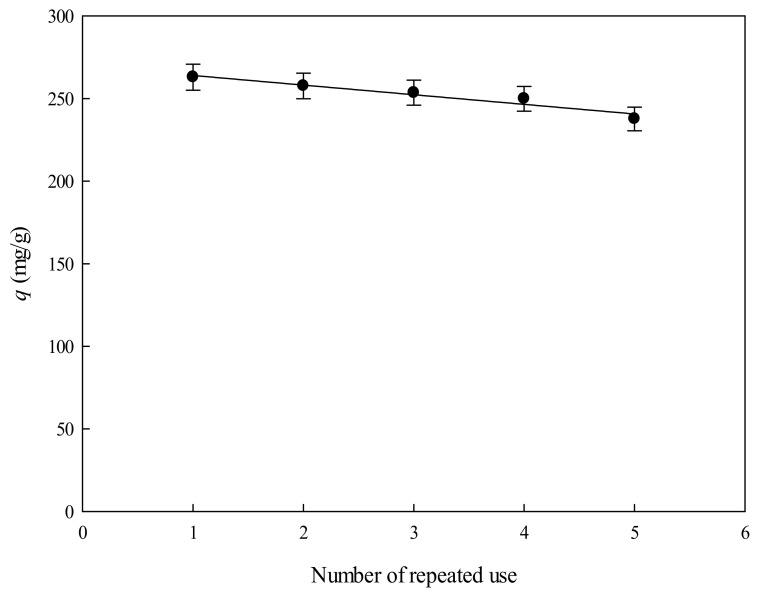
Effect of number of the repeated use of the P-EDA-Dye nanofiber membrane on the binding capacity of lysozyme by P-EDA-Dye nanofiber membrane.

The possibility of reusing P-EDA-Dye nanofiber membrane for multiple cycles of lysozyme adsorption was explored in this study. The adsorption–desorption operations in batch mode were repeated for five cycles using the same P-EDA-Dye nanofiber membrane as shown in Figure 10. The time of cleaning-in-place (CIP) procedure was carried for 1 h using a cocktail solution composed on 1 M NaCl, 25% ethanol, and 6 M urea. The P-EDA-Dye nanofiber membrane was successfully cleaned, as indicated by its binding capacity for lysozyme being not markedly reduced (<5%). Our results indicated that the CIP procedure used for the P-EDA-Dye nanofiber membrane was very effective.

## 4. Conclusions

The kinetics of adsorption of lysozyme onto the P-EDA-Dye nanofiber membrane was studied experimentally and theoretically under different operating conditions. The values of kinetic parameters associated with the adsorption mechanism of lysozyme were determined in a batch stirred tank system. The results showed that the pseudo second-order kinetic model gave the best correlation of the experimental data for all operating conditions used in this study. The surface reaction was proposed to be the rate-limiting step for the adsorption of lysozyme onto the membrane. Moreover, the pseudo second-order kinetic model could estimate the maximum binding capacity for lysozyme, since the *q_eq_* values are difficult to be determined experimentally. From our experimental data, the activation energy for lysozyme adsorption onto the P-EDA-Dye nanofiber membrane was determined to be ~2389.37 cal/mol at the temperature range of 278–308 K. The isotherm curves correlated well with the Langmuir model. The binding capacity decreased with increasing buffer ionic strength. This may be due to the presence of electrostatic interaction between dyed membrane and lysozyme. The interaction may play an important role in the adsorption process of lysozyme. In addition, our results showed that the values of Δ*G*° were negative, indicating the adsorption process to be a spontaneous reaction. The negative value of Δ*H*° showed that the adsorption was an exothermic process and the positive values of Δ*S*° indicated an increase randomness at the solid–liquid interface during lysozyme adsorption. Further research will be needed to demonstrate that CB dyes immobilized on the nanofiber membrane may serve as an alternative technique for the purification of lysozyme by membrane adsorption chromatography.

## Figures and Tables

**Table 1:** Kinetic constants calculated from pseudo first-order, pseudo second-order, Elovich, and intraparticle models for the adsorption of lysozyme by P-EDA-Dye nanofiber membrane.

Kinetic Models	Lysozyme (mg/mL)			NaCl (M)			Temperature (K)			
	0.1	0.5	1.0	0.1	0.2	1.0	278	288	298	308
*q_e,exp_* (mg/g)	35.43	214.31	266.18	205.72	137.26	35.82	235.96	259.55	266.18	373.73
Pseudo first-order model										
*k*_1_	0.1232	0.1003	0.0698	0.1365	0.0617	0.0407	0.0545	0.0542	0.0698	0.0708
*R* ^2^	0.8991	0.9732	0.9827	0.9893	0.9985	0.8357	0.8093	0.9689	0.9806	0.9747
Pseudo second-order model										
*k* ^2^	0.0541	4.02 × 10^−4^	3.54 × 10^−4^	1.66 × 10^−3^	1.38 × 10^−3^	0.0121	2.67 × 10^−4^	3.55 × 10^−4^	3.54 × 10^−4^	3.71 × 10^−4^
*q_e,cal_* (mg/g)	35.79	235.29	298.69	217.11	145.52	35.74	268.89	286.95	298.69	402.42
*R* ^2^	0.9999	0.9662	0.9807	0.9966	0.9902	0.9898	0.9716	0.9779	0.9827	0.9922
Elovich model										
α	59.23	46.96	56.89	94.02	47.14	35.88	43.37	56.41	56.89	126.72
*β*	0.1566	0.0174	0.0150	0.0207	0.0317	0.1588	0.0182	0.0165	0.0150	0.0127
*R* ^2^	0.6267	0.9821	0.9742	0.9275	0.9756	0.7133	0.9792	0.9742	0.9776	0.9792
Intra-particle diffusion model										
*k_i_*_1_	14.89	44.43	48.10	50.62	35.12	16.75	30.99	36.24	50.97	53.48
*R* ^2^	0.9958	0.9970	0.9941	0.9984	1.0	1.0	0.9936	0.9713	0.9945	0.9959
*k_i_*_2_	-	21.62	14.44	1.21	17.75	-	23.60	23.54	31.01	24.44
*R* ^2^	-	0.9911	0.8881	1.0	0.9920	-	0.9695	0.9930	0.9973	0.9644

**Table 2:** Comparison of *R*^2^ values for Langmuir, Freundlich, and Temkin isotherms at different temperatures.

Temperature (K)	Langmuir	Freundlich	Temkin
308	0.9983	0.8658	0.9475
298	0.9945	0.8541	0.8918
288	0.9955	0.9323	0.9725
278	0.9949	0.9836	0.9878

**Table 3:** Thermodynamic parameters calculated from Langmuir model for lysozyme adsorption by P-EDA-Dye nanofiber membrane at different temperatures.

Temperature (K)	*q_max,exp_* (mg/g)	*q_max,cal_* (mg/g)	*K_d_* (mg/mL)	*K_L_*(mL/mg)	*R^2^*	$\Delta G_{}^{{}^{\circ}}\;(\mathrm{cal}/\mathrm{mol})$	$\Delta H_{}^{{}^{\circ}}\;(\mathrm{cal}/\mathrm{mol})$	$\Delta S_{}^{{}^{\circ}}\;(\mathrm{cal}/\mathrm{mol}\cdot k)$
308	373.20	407.66	0.0506	19.15	0.9983	−1806.80	3739.93	18.01
298	275.50	297.09	0.0612	16.33	0.9945	−1653.81		18.10
288	256.36	282.65	0.0845	11.83	0.9955	−1413.84		17.90
278	231.73	262.19	0.0792	12.63	0.9949	−1395.85		18.48

## Data Availability

The data presented in this study are available on request from the corresponding author.
